# Selective aptamer conjugation to silver-coated magnetite nanoparticles for magnetic solid-phase extraction of trace amounts of Pb^2+^ ions

**DOI:** 10.1039/d1ra00006c

**Published:** 2021-01-27

**Authors:** Sara Rahnama, Shahab Shariati, Faten Divsar

**Affiliations:** Department of Chemistry, Rasht Branch, Islamic Azad University Rasht Iran shariaty@iaurasht.ac.ir +98-13-33447060 +98-13-3342-3153; Department of Chemistry, Payame Noor University PO Box 19395-3697 Tehran Iran

## Abstract

Herein, a novel aptamer-functionalized magnetic adsorbent was developed and combined with magnetic solid-phase extraction (MSPE) for the specific enrichment of Pb^2+^ ions prior to flame atomic absorption spectrometric detection. First, silver-coated magnetite core–shell nanoparticles (Fe_3_O_4_@Ag MNPs) were synthesized by the chemical reduction of silver ions on the surface of magnetite nanoparticles. After that, the selective DNA aptamer against Pb^2+^ was conjugated on the surface of the synthesized nanoparticles to form aptamer-modified magnetic nanoparticles (Fe_3_O_4_@Ag-APT). The characterization of the prepared adsorbent was performed through SEM imaging, XRD, FT-IR, EDX, and DRS instruments. The influence of the various experimental parameters on the adsorption and desorption steps in MSPE was investigated *via* Taguchi experimental design to optimize different parameters. Under the optimized conditions, the Pb^2+^ calibration graph was linear in the range of 33–1000 μg L^−1^. The relative standard deviation (RSD%) of the method for six replicates containing 100 μg L^−1^ of Pb^2+^ ions was 0.34%. Furthermore, the limit of detection (LOD) and the limit of quantification (LOQ) were 10 μg L^−1^ and 33.3 μg L^−1^, respectively. Finally, the applicability of the proposed method was successfully confirmed by preconcentration and determination of trace amounts of Pb^2+^ ions in tap and seawater samples. We showed a proof of concept for Fe_3_O_4_@Ag-APT as an efficient bio-adsorbent, offering a promising strategy for the specific binding/removal of toxic heavy metal ions.

## Abstract 2

Herein, a novel aptamer-functionalized magnetic adsorbent was developed and combined with magnetic solid-phase extraction (MSPE) for the specific enrichment of Pb^2+^ ions prior to flame atomic absorption spectrometric detection.

## Graphical abstract

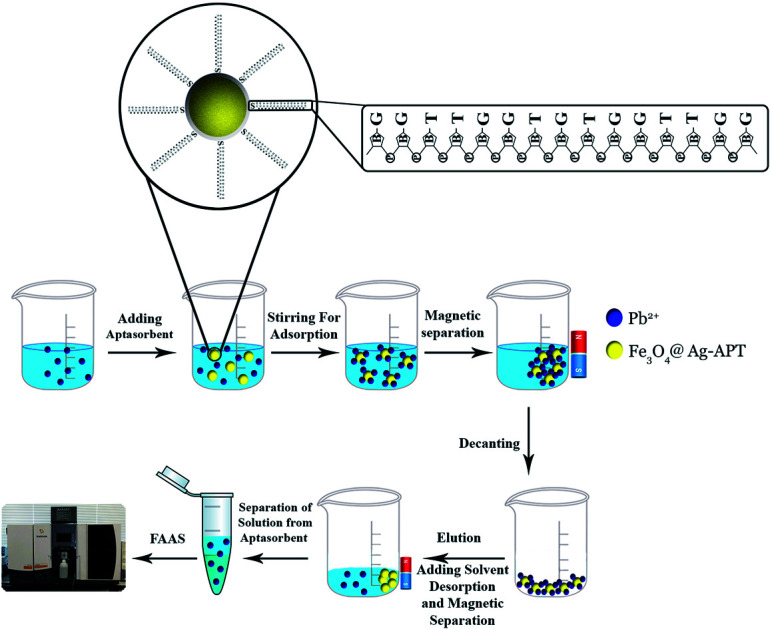

## 1. Introduction

One of the most serious ecological problems affecting the quality of water is chemical pollution by inorganic or organic compounds, including heavy metals, pharmaceuticals, dyes, drugs, pesticides and biocides.^1^ Various toxic heavy metals can accumulate in water and different food chains, such as human bodies *via* food, drinking water, breathing airborne particles and road dust .^2^ Therefore, the continuous monitoring of trace levels of metal ions in the environment is of major importance. Among the inorganic pollutants, lead is highly toxic owing to its accumulative toxicity to humans and animals. At high exposure levels in blood, it can affect human health by causing coma, convulsion, anemia, damage to organs, such as brains, bones, kidneys, and muscles, and also many serious illnesses or even mortality.^3,4^ Atomic absorption spectrometry (AAS) is known as the common instrumental method for the determination of heavy metals. However, the conventional determination of metal ions at low concentration by flame atomic absorption spectrometry (FAAS) is frequently not possible because their concentrations in water samples are typically at μg L^−1^ levels and below the detection limit of FAAS. To solve this problem, preconcentration of an analyte and/or separation procedures coupled with FAAS have been proposed.^5–9^

Solid-phase extraction (SPE) is the most popular pre-treatment method for sample matrix simplification and the enrichment of trace amounts of analyte in samples. SPE has become a strong method compared to preparation techniques such as liquid–liquid extraction, floatation, membrane filtration, and cloud point extraction because of the advantages such as high reproducibility, simplicity, low cost, lower consumption of organic solvents, high enrichment factor and applicability in different formats for various organic or inorganic analytes. The ability to be coupled with different detection techniques and a wide variety of sorbents are some of the benefits of SPE.^10–12^ Several types of adsorbent, such as mesoporous materials, activated carbon, ion-imprinted and carbon nanotubes, resins and nanoparticles, have been used in SPE.^13,14^ Among these sorbents, magnetic nanoparticles have attracted great interest because the magnetic separation shows excellent ability for the collection of magnetic nanomaterials from sample solutions by using an external magnetic field.^15–21^

Over the last decade, it has become popular for detection systems to use DNA for the detection of metal ions, mainly because DNA has good stability, is highly soluble in aqueous solution, and is a specific binder of some metal ions.^22,23^

Aptamers are short single-stranded oligonucleotides, either DNA or RNA (ssDNA or ssRNA), with affinity and specificity for the recognition, separation and detection of target compounds, namely peptides, toxins, proteins, drugs, microbes, organic and inorganic molecules, metal ions or even entire cells.^24,25^ Aptamers can be natural or synthetic in origin. They are isolated and chemically synthesized using combinatorial enrichment (SELEX).^26,27^ Aptamers bind constantly to a specific molecular target and as a result of their specific three-dimensional (3D) structure they can form particular complexes with the target molecule, which is a powerful tool for the identification of the goal molecules.^28–30^ Aptamers have been used in SPE for the enrichment, purification and extraction of particular analytes.^31,32^ The great merit of extraction using aptamers is the high selectivity, simple analytical process, extraction recovery, and fast processing. Because of the numerous advantages of aptamers, such as easy and economical synthesis and chemical modification, excellent stability against temperature and pressure variations, high affinity and specificity, small size, and reversible denaturation,^33^ they can be used for conjugating/modifying nanomaterials for the detection and diagnosis of heavy metals, proteins, and cancer cells.^34–36^ Recently, several strategies have been suggested for applying aptamers conjugated with nanomagnetic materials, including the use of aptamers combined with magnetic nanoparticles as modified adsorbents in magnetic solid-phase extraction (MSPE).^16,17,37,38^

The aim of this study was to develop a novel selective aptamer-based MSPE for the preconcentration and detection of Pb^2+^ ions by FAAS. First, Fe_3_O_4_ MNPs were synthesized *via* chemical precipitation method. The core–shell-structured Fe_3_O_4_@Ag MNPs were prepared by chemical reduction of silver ions on the surface of magnetite nanoparticles. The 15-mer DNA aptamer selective for Pb^2+^ ions^39,40^ was immobilized on the surface of the Fe_3_O_4_@Ag MNPs, giving the aptamer-based MSPE (APT-MSPE) sorbent. The influence of various experimental factors on the adsorption and preconcentration of Pb^2+^, including pH of solution, contact time, amount of nanoparticles, volume of aptamer, type and volume of elution solvent, concentration of elution solvent and time of desorption, was investigated *via* Taguchi experimental design method to optimize the different parameters. The prepared Fe_3_O_4_@Ag-APT sorbent was evaluated in terms of selectivity, binding capacity and extraction ability.

## 2. Materials and methods

### 2.1. Chemicals and reagents

All of the chemicals used, including ferrous chloride tetrahydrate (FeCl_2_·4H_2_O), ferric chloride hexahydrate (FeCl_3_·6H_2_O), ammonia solution (28%, w/w), hydrochloric acid (37%, w/w), silver nitrate (AgNO_3_), sodium borohydride (NaBH_4_), lead(ii) nitrate (Pb(NO_3_)_2_), cadmium nitrate tetrahydrate (Cd(NO_3_)_2_·4H_2_O), iron(iii) nitrate nonahydrate (Fe(NO_3_)_3_·9H_2_O), nickel(ii) nitrate hexahydrate (Ni(NO_3_)_2_·6H_2_O), cobalt(ii) nitrate hexahydrate (Co(NO_3_)_2_·6H_2_O), chromium(iii) nitrate nonahydrate (Cr(NO_3_)_3_·9H_2_O), sodium nitrate (NaNO_3_), potassium nitrate (KNO_3_), lithium nitrate (LiNO_3_), copper(ii) nitrate trihydrate (Cu(NO_3_)_2_·3H_2_O), aluminum nitrate nonahydrate (Al(NO_3_)_3_·9H_2_O), calcium nitrate tetrahydrate (Ca(NO_3_)_2_·4H_2_O), barium nitrate (Ba(NO_3_)_2_), and zinc nitrate tetrahydrate (Zn(NO_3_)_2_·4H_2_O) were prepared with the analytical reagent grade from Merck (Darmstadt, Germany), Fluka (Buchs, Switzerland) and Sigma-Aldrich (ST. Louise, Missouri, USA). The 5′-thiol-modified DNA oligonucleotide (5′-GGTTGGTGTGGTTGG-3′) for the aptamer was synthesized and purified by HPLC in Generay Biotech (Shanghai) Co. Ltd (China). This aptamer is a kind of oligonucleotide (single-stranded DNA) consisting of nine deoxyguanosine and six thymidine residues connected by 3′ → 5′ phosphodiester linkages in the sequence 5′-HS-G-G-T-T-G-G-T-G-T-G-G-T-T-G-G-3′ (C_150_H_188_N_57_O_97_P_15_, length: 15 nucleotides), that was modified with 5′ SH (MW: 4923.0 g mol^−1^). Phosphate-buffered saline (PBS, 0.1 M, pH 7.4) was prepared by dissolving NaH_2_PO_4_·2H_2_O, Na_2_HPO_4_·12H_2_O and KNO_3_ in double-distilled water and was used as a buffer for preparing the DNA solutions. A stock standard solution of lead ions (1000 mg L^−1^ of Pb^2+^) was prepared by dissolving pure lead(ii) nitrate salt in double-distilled water. The working solutions of Pb^2+^ ions were made up using successive dilutions in PBS.

### 2.2. Instruments and apparatus

The prepared Fe_3_O_4_@Ag nanoparticles (MNPs) were characterized by X-ray diffraction (XRD) using CuKα with 2*θ* values of 5–80°. Scanning electron microscopy and energy-dispersive X-ray spectroscopy (SEM and EDX, model LEO1430VP, England and Germany) were used to observe the surface morphology and for the elemental analysis of the magnetic nanoparticles. Fourier transform infrared (FT-IR) spectra of the synthesized MNPs were obtained using an FT-IR instrument from Shimadzu (model 8900, Japan) in the range of 400–4000 cm^−1^. The UV-vis diffuse reflectance spectroscopy (DRS) of the synthesized nanoparticles was recorded on a Scinco spectrophotometer (S-4100, South Korea) equipped with a DRS accessory. In this work, a flame atomic absorption spectrometer (FAAS) model AA240FS (Varian, USA) equipped with a deuterium lamp for background correction was used for the determination of Pb^2+^ ions in an air–acetylene flame. The parameters for the FAAS detection of Pb^2+^ ions were as follows: hollow cathode lamp (HCL); wavelength, 217 nm; slit width, 1.0 nm; and lamp current, 10 mA. The pH of the solutions was measured using a Bante pH meter equipped with a combined glass electrode (China). For magnetic separation, a strong permanent magnet (1 × 3 × 5 cm) with a 1.4 T magnetic field was applied. A magnetic stirrer (Labinco, The Netherlands) and stirrer bar (4 × 14 mm) were used in the nanoparticle synthesis.

### 2.3. Preparation of aptamer-based silver-coated magnetite nanoparticles (Fe_3_O_4_@Ag-APT MNPs)

#### 2.3.1. Synthesis of Fe_3_O_4_@Ag core–shell nanostructures

Fe_3_O_4_ MNPs were chemically prepared according to a method described in the literature.^41^ Briefly, FeCl_3_·6H_2_O and FeCl_2_·4H_2_O (molar ratio of 2 : 1) and 1.5 mL of HCl (12 mol L^−1^) were dissolved in 50 mL of double-distilled water in a volumetric flask to prepare the stock solution of ferric and ferrous chloride. Then, the clear yellow solution was degassed with nitrogen gas for 10 min. Simultaneously, 250 mL of ammonia solution (4.5 mol L^−1^) was degassed with nitrogen gas and heated to 80 °C in a reactor. After that, the mixture of ferric and ferrous ions was added to the ammonia solution slowly *via* a dropping funnel with vigorous stirring (1000 rpm) under gas protection for 30 min. During the whole process, the temperature of the solution was held at 80 °C and nitrogen gas was purged to remove the dissolved oxygen. After the formation of the black magnetic nanoparticles (Fe_3_O_4_ MNPs), they were separated from the reaction medium using an external magnet and washed with double-distilled water and sodium hydroxide solution (0.1 mol L^−1^).

The Fe_3_O_4_@Ag MNPs were prepared by chemical reduction of silver nitrate on the surface of the MNPs in advance using the following modified protocol.^42^ Briefly, 1 g of Fe_3_O_4_ MNPs was dispersed in 50 mL of double-distilled water, followed by the addition of 25 mL of AgNO_3_ (0.001 mol L^−1^) under vigorous stirring. After 30 min, 50 mL of 3 × 10^−4^ mol L^−1^ NaBH_4_, as a reductant, was added dropwise to the suspension under vigorous mechanical stirring for another 30 min. After this time, the green core–shell nanoparticles were collected using a permanent magnet, washed several times with double-distilled water to remove excess Ag particles and then dried at 70 °C.

#### 2.3.2. Immobilization of the aptamer on the magnetic nanoparticles

The purchased aptamer sample was centrifuged at 10 000 rpm (2 min) to settle the aptamer at the bottom of the vial. The stock solution of the thiolated lead-binding aptamer (100 μM) was prepared *via* addition of 123 μL of 0.1 mol L^−1^ PBS (pH 7.4) to the aptamer vial. After vortexing (1 min), the mixture was kept at −20 °C. Aptamer solutions with concentrations of 10 and 5 μM were prepared by diluting the aptamer stock solution (100 μM) with 0.1 mol L^−1^ PBS solution and vortexing (1 min); the solutions were then stored at −20 °C.

**Fig. 1:**
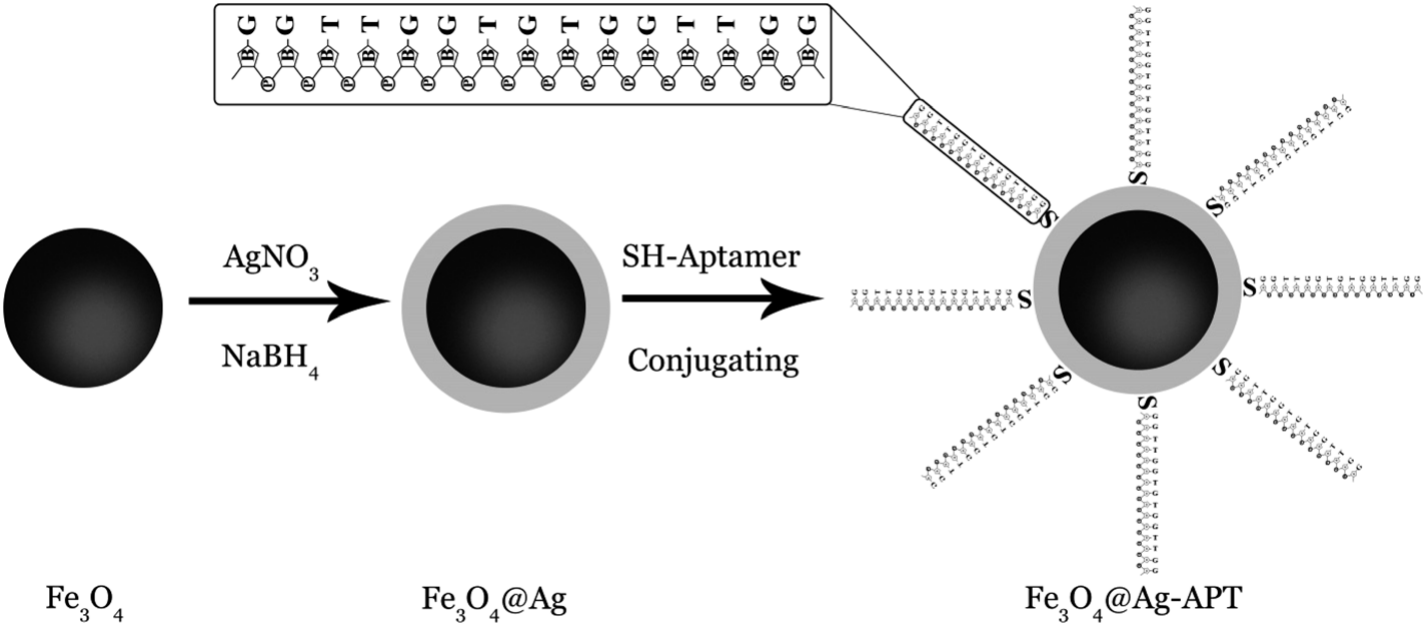
Schematic illustration of the fabrication of Fe_3_O_4_@Ag-APT.

For synthesis of the Fe_3_O_4_@Ag-APT MNPs, the volume of aptamer solution and the weight of sorbent were optimized by Taguchi experimental design. For the synthesis, 0.04 g of Fe_3_O_4_@Ag MNPs was weighed and transferred into a 10 mL beaker. After addition of 1 mL of PBS, the suspension was sonicated (5 min) to prevent aggregation of the MNPs. A certain volume of 5 μM aptamer (optimum: 45 μL) solution was added to the suspension. The solution was stirred for 24 h. After this time, the synthesized Fe_3_O_4_@Ag-APT MNPs were separated using a super magnet and the supernatant was disposed of. Fig. 1 shows a schematic of the synthesis procedure.

#### 2.3.3. Magnetic solid-phase extraction of Pb^2+^ ions using Fe_3_O_4_@Ag-APT sorbent

**Fig. 2:**
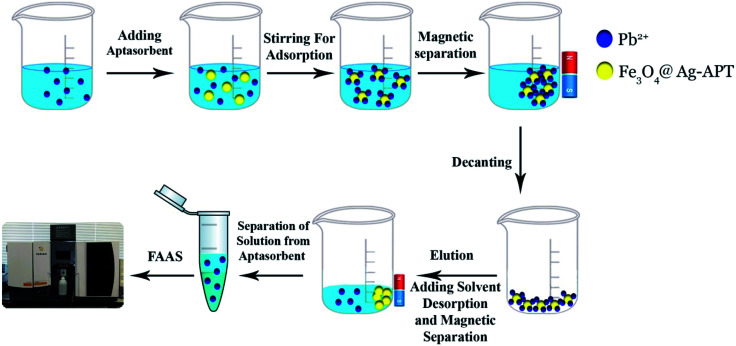
Schematic diagram of preconcentration of Pb^2+^ ions by aptamer.

For each MSPE experiment, 25 mL of an aqueous sample containing certain amounts of Pb^2+^ ions was poured into a beaker and, after adjusting the pH of the solution to 7.0, 0.04 g of Fe_3_O_4_@Ag-APT MNPs was added to the mixture. Afterwards, the mixture was mechanically stirred for 35 min at 400 rpm. After this time, the MNPs were isolated from the mixture using an external magnetic field by applying a strong super magnet. The supernatant was transferred to a vial to check for the presence of Pb^2+^. The preconcentrated ions were desorbed from the collected MNPs during stirring with 3 mL of 0.25 mol L^−1^ HCl (10 min). After that, the solution containing the eluted Pb^2+^ ions was collected in a vial for further measurement by FAAS (Fig. 2).

## 3. Results and discussion

### 3.1. Principle of the MSPE strategy using Fe_3_O_4_@Ag-APT MNPs

Metal coordination by DNA has been extensively studied.^43–45^ DNA is a polyanion allowing electrostatic attraction with metal ions. DNA phosphates can bind hard/borderline metals, while various bases coordinate with metal ions with different affinities. At the simplest level, metal ions are treated as point charges diffusing around DNA polyanions by pure electrostatic interactions. Metal binding to the phosphate backbone stabilizes the DNA duplex (*e.g.*, increasing DNA melting temperature, Tm). Pb^2+^-dependent G-rich ssDNAs can be integrated into Pb^2+^ biosensors. Upon addition of Pb^2+^ ions to the solution, the conformation of the Pb^2+^-dependent aptamer changes from a random coil structure to a G-quadruplex one.

### 3.2. Characterization of Fe_3_O_4_@Ag MNPs

The Fe_3_O_4_@Ag MNPs were characterized using XRD, SEM, EDX, FT-IR and DRS analysis.

**Fig. 3:**
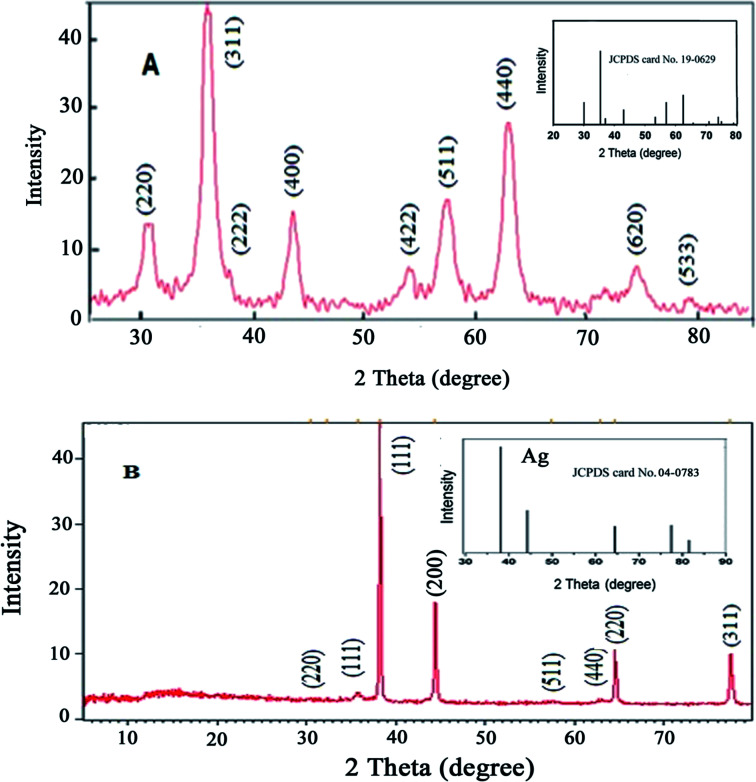
XRD patterns for (A) Fe_3_O_4_ and (B) Fe_3_O_4_@Ag MNPs.

The crystal phases and crystallinity of Fe_3_O_4_ and Fe_3_O_4_@Ag MNPs were analyzed by XRD and measured with Cu Kα radiation in the 2*θ* values, of 5–80°, with a step size of 0.0260° and count time of 0.5°/s. According to the results (Fig. 3(a and b)), the characteristic peaks at 2*θ* of 30.3° (220), 35.5° (311), 37° (222), 43.3° (400), 54° (422), 57° (511), 63° (440) and 74.5° (533) show the crystalline cubic spinel structure of Fe_3_O_4_ (Fig. 3(a)), which is in agreement with the standard of Fe_3_O_4_ (JCPDS No: 19-0629) and previous literature.^46^

The absence of Fe_2_O_3_ peaks in the 2*θ* range of 20–30° provided proof that the black synthesized powder is Fe_3_O_4_. The characteristic peaks presented at 2*θ* values of 38.2° (111), 44.4° (200), 64.6° (220) and 77.5° (311) are related to the planes of silver (Fig. 3(b)), in agreement with the XRD pattern of Ag NPs (JCPDS No: 04-0783). The XRD patterns confirmed that no impurities were observed.

**Fig. 4:**
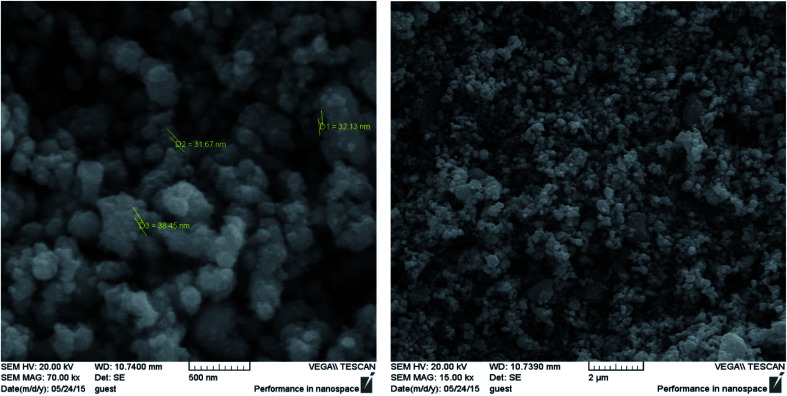
SEM images of Fe_3_O_4_@Ag MNPs.

SEM observations were performed to characterize the morphology and size of the synthesized Fe_3_O_4_@Ag MNPs with magnification of 15.0 and 70.0kx (Fig. 4). Based on the SEM images, the synthesized Fe_3_O_4_@Ag MNPs were uniform spherical particles with an average diameter of less than 38 nm. The nanoparticles could provide a large specific surface area and numerous adsorption sites. Moreover, the addition of the aptamer did not alter the morphology or particle size of the nanoparticles distinctly.

**Fig. 5:**
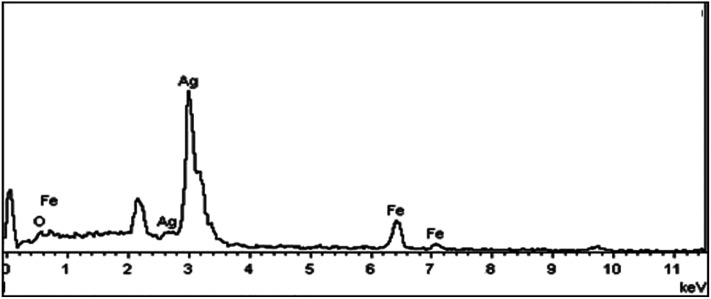
EDX spectrum for Fe_3_O_4_@Ag MNPs.

Elemental analysis was also used to confirm the existence of silver element on the Fe_3_O_4_ nanoparticles. The atomic composition of the Fe_3_O_4_@Ag was evaluated by EDX analysis. Fig. 5 presents the EDX spectrum of Fe_3_O_4_@Ag MNPs, where peaks associated with Fe, Ag and O can be distinguished. Quantitative analysis gave weight ratios of Fe (16.80%), Ag (35.20%) and O (47.99%), as previously reported.^47^

**Fig. 6:**
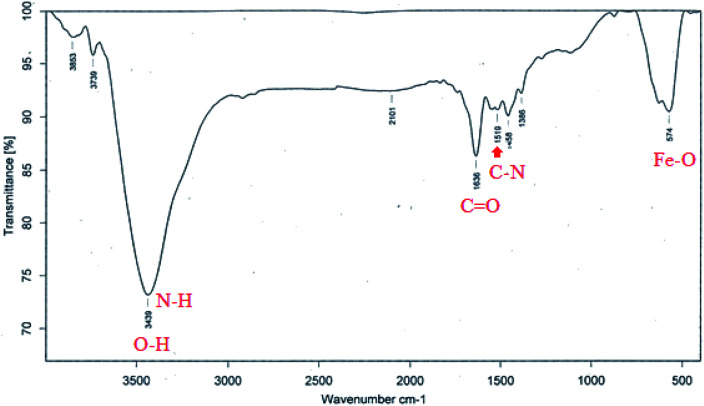
FT-IR spectrum for Fe_3_O_4_@Ag-APT MNPs.

Fig. 6 shows the FT-IR spectrum of Fe_3_O_4_@Ag-APT MNPs. In this spectrum, the band at 574 cm^−1^ corresponds to the Fe–O stretching vibration mode of the tetrahedral and octahedral sites. The absorption bands centered at 1519 and 1636 cm^−1^ could be attributed to the stretching vibration of the C–N and C=O bonds of peptide linkages, respectively. The wide band at 3439 cm^−1^ could be assigned to the O–H stretching mode of the adsorbed water and N–H bending mode.^48^

**Fig. 7:**
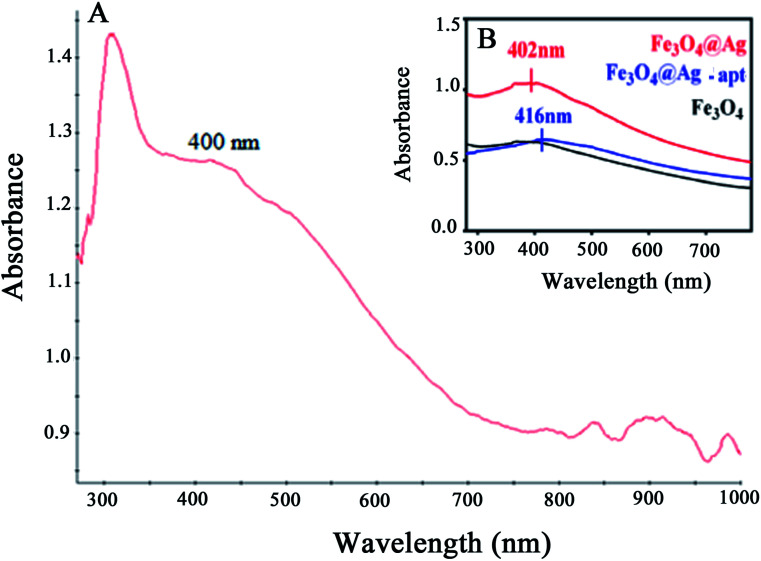
UV-Vis absorption spectra for (A) Fe_3_O_4_@Ag MNPs and (B) Fe_3_O_4_, Fe_3_O_4_@Ag and Fe_3_O_4_@Ag-Apt MNPs.

The UV-Vis absorption spectra of the Fe_3_O_4_@Ag MNPs, Fe_3_O_4_ and Fe_3_O_4_@Ag-Apt MNPs are shown and compared in Fig. 7. The absorbance of Fe_3_O_4_ decreases with increasing wavelength in the range 350–800 nm, which agrees well with the literature,^49^ and no obvious absorption peak was observed. The Fe_3_O_4_@Ag MNPs have an absorption peak at around 400 nm resulting from the typical surface plasmon resonance of silver NPs. This result shows that silver was successfully deposited on the surface of the Fe_3_O_4_ MNPs.

### 3.3. Optimization of the MSPE adsorption parameters

The 5′-thiolated aptamer was attached to the Ag layer of the Fe_3_O_4_@Ag MNPs to form an aptamer-based sorbent. The Pb^2+^ ions reacted with the functional groups of the oligonucleotide present in the structure of the aptamer and were separated from the solution.

**Fig. 8:**
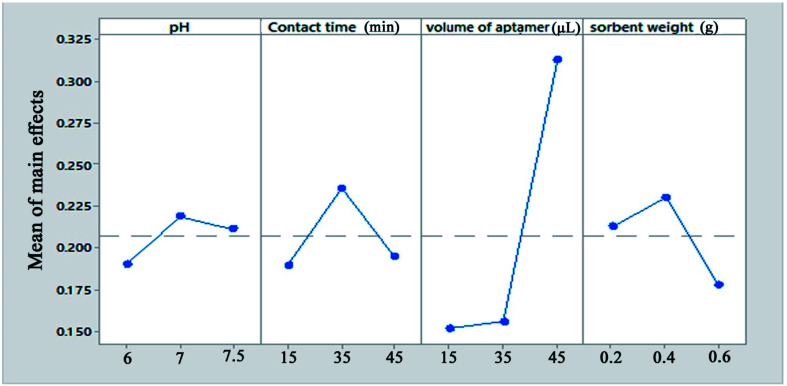
The means of the main effects of each factor in the adsorption process obtained by Minitab software.

For the MSPE optimization experiments, 25 mL of a solution containing 1 mg L^−1^ Pb^2+^ was exposed at room temperature with the proper amount of sorbent. After magnetic separation, the Pb^2+^ adsorption and desorption efficiencies were determined using FAAS (Fig. 2). In preliminary tests, the effect of the volume of aptamer solution used in the synthesis of the sorbent was investigated. For this purpose, 2, 5, 10, 15 and 20 μL of 5 μM aptamer solution were used for 0.02 g of Fe_3_O_4_@Ag MNPs to produce the aptamer-based sorbent. According to the preliminary tests, at aptamer volumes lower than 15 μL, the aptamer did not attach to the MNPs properly. Therefore, in the optimization table, aptamer volumes of 15 μL and higher were considered. All of the parameters affecting the adsorption and desorption of Pb^2+^ ions on the sorbent were optimized separately *via* Taguchi orthogonal optimization to obtain the highest sensitivity of sorbent. To fully develop the aptamer-based sorbent for analysis of Pb^2+^ ions, some significant adsorption factors including the pH of the solution (6.0, 7.0, 7.5), contact time (15, 35, 45 min), sorbent weight (0.02, 0.04, 0.06 g) in solution and volume of aptamer solution (15, 35, 45 μL) were investigated and optimized *via* Taguchi orthogonal array design using an L_9_ array (4 factors at 3 levels, Table 1). After carrying out the Taguchi design optimization experiments, statistical calculations were performed and the average main effects of each factor were obtained at different levels using Minitab software (version 18) (Fig. 8). Based on the results of the experiments and the analysis of variance (ANOVA), the optimal conditions for the adsorption of Pb^2+^ ions were obtained according to the mean main effects for each parameter (Fig. 8).

**Table 1:** The OA_9_ Taguchi method for optimization the experimental factors

Ex. no	Aptasorbent weight (g)	Aptamer volume (μL)	Contact time (min)	pH
1	0.04	45	15	7
2	0.06	45	35	6
3	0.02	45	45	7.5
4	0.02	15	15	6
5	0.04	15	35	7.5
6	0.02	35	35	7
7	0.04	35	45	6
8	0.06	15	45	7
9	0.06	35	15	7.5

Solution pH is an important parameter affecting the efficiency of Pb^2+^ ion adsorption from aqueous samples. pH plays an important role in the complexation of Pb^2+^ ions with the aptamer. In this study, the solution pH was varied between 6 and 7.5 (near the physiological pH). According to the results, the maximum Pb^2+^ adsorption efficiency was observed at pH = 7.0. According to the results, at physiological pH, the synthesized aptamer is extremely stable and has a negative charge on its surface. Therefore, a pH of 7.0 was selected for further studies.

The maximum adsorption efficiency was obtained with 0.04 g of aptamer, which can be attributed to the increase in the number of sorbent sites in the solution. However, the extraction efficiency decreased when more than 0.04 g of Fe_3_O_4_@Ag MNPs was used. This is due to that a small quantity of Fe_3_O_4_@Ag MNPs could not be dispersed due to hydrophobic surface of it or attachment of Fe_3_O_4_@Ag MNPs onto magnet. This hindered the collection of all of the sorbent by an external magnet, which resulted in a slight decrease in the extraction efficiency. Based on the results, 0.04 g of sorbent provided the highest extraction efficiency for all analytes.

Moreover, the amount of lead ions bound to the Fe_3_O_4_@Ag-APT is a function of the aptamer surface density, which clearly justifies the need for optimization. Therefore, the different amounts of the thiolated DNA aptamer were added to 1 mL of a suspension containing 0.04 g of Fe_3_O_4_@Ag in PBS and then the aptamers were anchored onto the surface of the magnetic nanoparticles *via* immobilization by thiol chemistry. It was found that a low aptamer density on the surface, which affects the amount of target binding, leads to low signal levels. However, the maximum amount of target bound is a function of the aptamer surface density. The aptamer surface density increased and reached a maximum with 45 μL of aptamer.

In the case of the contact time, the majority of lead ions were captured by aptamer-modified Fe_3_O_4_@Ag MNPs within 20 min and the maximum recovery for Pb^2+^ ions was obtained within 35 min. However, a long stirring time may cause partial release of adsorbed Pb^2+^ ions from the sorbent.

### 3.4. Optimization of Pb^2+^ desorption parameters affecting the MSPE procedure

After adsorption of Pb^2+^ ions using Fe_3_O_4_@Ag-APT MNPs and prior to their measurement by FAAS, it is necessary to desorb the adsorbed ions using the minimum volume of suitable eluent. For this purpose, several desorption parameters, including type of eluent (HCl in distilled water, HCl in phosphate buffer and HCl in ethanol), eluent concentration (0.05, 0.1 and 0.25 mol L^−1^), volume of eluent (3, 5 and 10 mL) and time of desorption (5, 10 and 15 min) were investigated *via* Taguchi experimental design using an L_9_ array (4 factors at 3 levels, Table 2) to achieve the best Pb^2+^ ion desorption efficiency. The desorption optimization experiments were carried out in the optimal conditions of adsorption process (pH = 7.0, sorbent weight = 0.04 g, contact time = 35 min and volume of aptamer = 45 μL).

**Table 2:** The OA_9_ Taguchi method for optimization of the desorption parameters^a^

Ex. no	Type of eluent	Eluent volume (mL)	Desorption time (min)	Eluent concentration (mol L^−1^)
1	HCl in DW	10	5	0.1
2	HCl in EtOH	10	10	0.05
3	HCl in PBS	10	15	0.25
4	HCl in PBS	3	5	0.05
5	HCl in DW	3	10	0.25
6	HCl in PBS	5	10	0.1
7	HCl in DW	5	15	0.05
8	HCl in EtOH	3	15	0.1
9	HCl in EtOH	5	5	0.25

a DW: distilled water; PBS: phosphate-buffered saline (0.1 mol L^−1^); EtOH: ethanol.

**Fig. 9:**
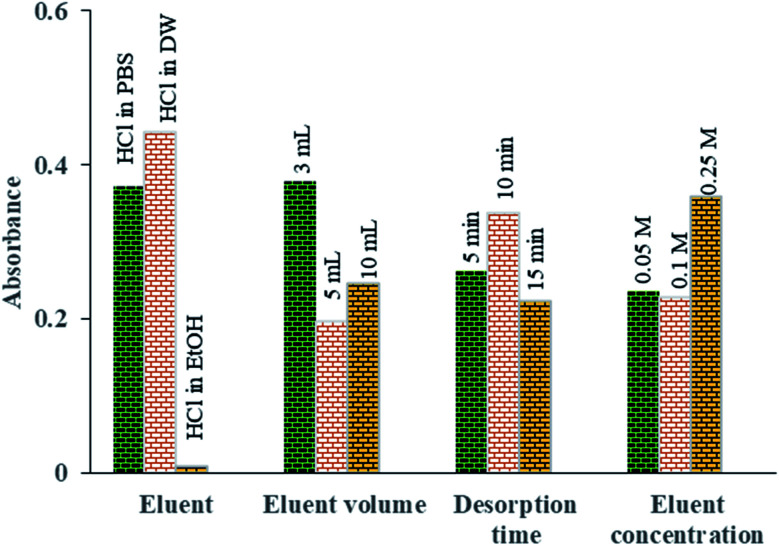
The means of the main effects of each factor in optimization of the desorption parameters obtained by absorbance reading of UV-Vis measurements.

To find the best eluent type to desorb the Pb^2+^ ions from the aptamer, HCl in three different solvents was investigated (distilled water, ethanol (EtOH) and PBS (0.1 mol L^−1^)). Among them, HCl in DW was found to be superior for the desorption of Pb^2+^ ions from the aptamer surface (Fig. 9).

Fig. 9 shows the effect of eluent volume (3 to 10 mL) on the amount of Pb^2+^ desorbed. The results indicate that by increasing the volume of the eluent, the Pb^2+^ absorbance and therefore its concentration in desorbed solution are reduced owing to the dilution of the eluent phase. Thus, to increase the preconcentration factor, 3 mL of eluent was considered to be the optimal eluent volume.

In order to investigate the effect of the concentration of the eluent solvent on the preconcentration of Pb^2+^ ions, different HCl concentrations were investigated in the range of 0.05 to 0.25 M. Based on Fig. 9, quantitative recovery of Pb^2+^ ions could be obtained with eluent containing 0.25 M HCl.

To achieve the highest preconcentration and optimal recovery of the adsorbed Pb^2+^ ions, the effect of desorption time was also tested between 5 and 15 min. The results showed that the maximum desorption efficiency of Pb^2+^ ions was obtained with 10 min of shaking of aptamer in 3 mL of HCl (0.25 mol L^−1^) (Fig. 9).

### 3.5. Analytical figures of merit

Under the optimized adsorption and desorption conditions (pH = 7.0, sorbent weight = 0.04 g, volume of aptamer = 45 μL, contact time = 35 min, desorption time = 10 min with 3 mL HCl in distilled water (0.25 M)), several analytical characteristics of the developed method were investigated, including linear dynamic range (LDR), limit of detection (LOD), limit of quantitation (LOQ) and enrichment factor (EF). The calibration curve was obtained without and after preconcentration of Pb^2+^ ions. The linear range for direct measurement without preconcentration was found to be 300–1500 μg L^−1^ with a correlation coefficient (*R*^2^) of 0.9991 while the LDR after preconcentration was observed to be in the range of 33–1000 μg L^−1^ with *R*^2^ = 0.9972. The limit of detection (3 *S*_b_/*m*) and limit of quantitation (10 *S*_b_/*m*) were 10 μg L^−1^ and 33.0 μg L^−1^, respectively. In these equations, *m* is the slope of the calibration graph after preconcentration and *S*_b_ is the standard deviation of four independent replicate preconcentration of blank solution. The precision of the method was assessed as relative standard deviation (RSD, %) and calculated from six independent replicates at a Pb^2+^ ion concentration of 100 μg L^−1^ and was found to be 0.34%. The EF of the proposed method, as the ratio of the slope of the calibration curve after preconcentration to that without preconcentration, was calculated as 17.37.

### 3.6. The effect of foreign ions on the MSPE of Pb^2+^ ions

An important feature of a sorbent is its selectivity towards the analyte over interfering matrix species. In order to evaluate the selectivity and the preconcentration efficiency of the prepared aptamer, various ions, such as Na^+^, Li^+^, K^+^, Ni^2+^, Cu^2+^, Co^2+^, Ca^2+^, Zn^2+^, Ba^2+^, Cd^2+^, Cr^3+^, Fe^3+^ and Al^3+^, were tested for their interference effects on the preconcentration of Pb^2+^ ions. Different concentrations of these ions were prepared in PBS and added to the test solution containing 100 μg L^−1^ of Pb^2+^ ions, then the developed method was applied. The tolerance limits for coexisting ions are summarized in Table 3. According to this table, the presence of foreign ions in solution with Pb^2+^ does not have an interfering effect on the preconcentration of Pb^2+^ ions.

**Table 3:** Tolerance limits for foreign ions in the presence of 100 μg L^−1^ Pb^2+^ ions

Foreign ion	Added as	Foreign ion concentration (mg L^−1^)	Foreign ion to metal ion ratio
Na^+^	NaNO_3_	100	1000
Li^+^	LiNO_3_	50	500
K^+^	KNO_3_	10	100
Ni^2+^	Ni(NO_3_)_2_·6H_2_O	50	500
Cu^2+^	Cu(NO_3_)_2_·3H_2_O	50	500
Co^2+^	Co(NO_3_)_2_·6H_2_O	10	100
Ca^2+^	Ca(NO_3_)_2_·4H_2_O	1	10
Zn^2+^	Zn(NO_3_)_2_·6H_2_O	5	50
Ba^2+^	Ba(NO_3_)_2_	50	500
Cd^2+^	Cd(NO_3_)_2_·4H_2_O	50	500
Cr^3+^	Cr(NO_3_)_3_·9H_2_O	50	500
Al^3+^	Al(NO_3_)_3_·9H_2_O	5	50
Fe^3+^	Fe(NO_3_)_3_·9H_2_O	50	500

### 3.7. Validation of the proposed MSPE method for analysis of real samples

In order to evaluate the analytical performance of the proposed aptamer, tap water (Tehran, Iran) and seawater (Caspian Sea, Gilan) were collected and investigated *via* the proposed MSPE method. Each sample was firstly analyzed for the presence of Pb^2+^ ions and the results showed no presence of this ion in the real samples. After that, each real sample was spiked with 100 μg L^−1^ of Pb^2+^ ions and the spiked samples were extracted using the proposed aptamer-based MSPE procedure. Three replicates were performed for all experiments. Pb^2+^ ion recoveries of 96.8 and 102.8% were obtained from the spiked sea and tap water samples, respectively, which were in good agreement with those obtained by ICP-OES. The recoveries for water samples tested by this assay are comparable with the amounts that have been previously reported in the literature,^50,51^ demonstrating the suitable accuracy of the proposed method for the preconcentration of Pb^2+^ ions.

Furthermore, under optimum conditions, the proposed procedure was applied for preconcentration and recovery of Pb^2+^ ions from contaminated aqueous solution using Fe_3_O_4_@Ag-APT MNPs and Fe_3_O_4_@Ag MNPs. The aptamer-modified MNPs displayed an excellent selectivity with higher affinity toward Pb^2+^ ions compared to that of the naked Fe_3_O_4_@Ag MNPs.

### 3.8. Comparison with other solid-phase extraction procedures in the literature

In 2015, Huang *et al.* used nitrilotriacetic acid anhydride-modified lignocellulosic material for removal of Cd^2+^ and Pb^2+^ from aqueous solutions. A sorption capacity of 303.5 mg g^−1^ was obtained for Pb^2+^ removal. The pseudo-second-order kinetic model and the Langmuir isotherm model described the adsorption process well. Thermodynamic data confirmed the endothermic and spontaneous sorption process.^52^ In 2016, Cheng *et al.* studied the biosorption of Pb^2+^ ions from aqueous solutions by waste biomass from biotrickling filters. The results showed that the maximum Pb^2+^ biosorption capacity of dried biomass was 160 mg g^−1^^53^

A comparison with other SPE adsorbents used for preconcentration of Pb^2+^ ions prior to FAAS is given in Table 4.^11,42,54–57^ Our proposed aptamer showed broad DLR and good LOD with respect to the other adsorbents. Moreover, by utilizing graphite furnace AAS (GF-AAS), ICP-AES or ICP-MS for the determination of Pb^2+^, the detection limit can be improved by more than 1000 times and it is possible to detect trace levels of Pb^2+^ ions. In addition, utilizing lower eluent volumes in GF-AAS improves the preconcentration factor.

**Table 4:** Comparison of the developed method with some extraction methods for preconcentration of Pb^2+^ ions

Metal ions	Sorbent	Sorbent mass (g)	Method	Detection method	LOD (μg L^−1^)	RSD (%)	LDR (μg L^−1^)	Time (min)	Ref.
Pb^2+^, Fe^3+^, Cu^2+^, Mn^2+^	Multiwall carbon nanotubes	0.1	SPE	FAAS	8.0	—	—	100	54
Pb^2+^	MnFe_2_O_4_-takovite	0.02	MSPE	FAAS	0.67	3	2–100	12	11
Pb^2+^, Cd^2+^	Silica gel	0.3	SPE	FAAS	4.25	1.7	—	10	55
Pb^2+^, Cu^2+^	Nanodiamond/MoS_2_ nanorod	0.25	SPE	FAAS	42	0.9	100–1000	6	56
Cd^2+^, Zn^2+^, Cu^2+^, Pb^2+^	Sulfur-nanoparticle-loaded alumina	0.5	SPE	FAAS	0.63	4.8	1–60	15	57
Pb^2+^	APT-MNPs	0.04	MSPE	FAAS	10	0.3	33–1000	10	This work

## 4. Conclusion

In this study, we reported the development of an aptasorbent Fe_3_O_4_@Ag-APT MNP for the selective extraction of Pb^2+^ ions. The introduced magnetic aptasorbent can be used as an easy, reliable, inexpensive, selective and sensitive method for the extraction and/or preconcentration of trace levels of lead ions in various aqueous matrices prior to determination. After carrying out Taguchi design optimization experiments, the best results for the adsorption and desorption of Pb^2+^ ions were obtained at pH 7.0 using 0.04 g of the sorbent modified with 45 μL of aptamer and a total contact time of 35 min for adsorption and 10 min for desorption, with 3 mL of HCl (0.25 mol L^−1^) as the eluent. Under the optimum conditions, Pb^2+^ was detected in the range of 33–1000 μg L^−1^ with a low detection limit of 10 μg L^−1^. It is noteworthy that, compared to previous works, this new protocol has several advantages. First, the sorbent is simpler than the previous ones; second, the results showed that the developed Fe_3_O_4_@Ag-APT exhibited a wide working range and impressively high selectivity toward Pb^2+^ among other heavy metal ions in aqueous media and also had good potential for the extraction of Pb^2+^ ions at μg L^−1^ concentrations.

## Conflicts of interest

There are no conflicts to declare.

## Supplementary Material

## References

[cit1] Dorival-Garci N., Zafra-Gornez A., Catarero S., Navalon A., Vilchez J. L. (2013). Simultaneous determination of 13 quinolone antibiotic derivatives in waste water sample using solid – phase extraction and ultra performance liquid chromatography-tandem mass spectrometry. Microchem. J..

[cit2] Ninwong B., Chuanuwatanakul S., Chailapakul O., Dungchai W., Motomizu S. (2012). On-line preconcentration and determination of lead and cadmium by sequential injection/anodic stripping voltammetry. Talanta.

[cit3] Chen L., Xu Z., Liu M., Huang Y., Fan R., Su Y., Hu G., Peng X. (2012). Lead exposure assessment from study near a lead – acid battery factory in china. Sci. Total Environ..

[cit4] Yavuz E., Tokalioglu S., Sahan H., Patat S. (2013). Ultralayered Co_3_O_4_ as a new adsorbent for preconcentration of Pb (ii) from water, food, sediment and tobacco samples. Talanta.

[cit5] Cui Y., Chang X., Zhu X., Luo H., Hu Z., Zou X. (2007). Chemically modified silica gel with p-dimethylaminobenzaldehyde for selective solid-phase extraction and preconcentration of Cr (iii), Cu (ii), Ni (ii), Pb (ii) and Zn (ii) by ICP-OES, Micro. Chem. J..

[cit6] Gana E. M., Lima A. D., Lemos V. A. (2006). Preconcentration system for cadmium and lead determination in environmental samples using polyurethane foam/Me-BTANC. J. Hazard. Mater..

[cit7] Meesri S., Praphairaksit N., Imyim A. (2007). Extraction and preconcentration of toxic metal ions from aqueous solution using benzothiazole-based chelating resins. Microchem. J..

[cit8] Refiker S. H., Merdivan M., Aygun R. S. (2008). Solid phase extraction of silver in geological samples and its determination by FAAS. Sep. Sci. Technol..

[cit9] Ferreira S. L. C., Lemos V., Santelli R. E. (2001). An automated on line flow system for the preconcentration and determination of lead by flame atomic absorption spectrometry, Micro. Chem. J..

[cit10] Huang C., Hu B. (2008). Silica-coated magnetic nanoparticles modified with γ-mercapto propyltrimethoxysilane for fast and selective solid phase extraction of trace amounts of Cd, Cu, Hg and Pb in environmental and biological samples prior to their determination by inductively coupled plasma mass spectrometry. Spectrochim. Acta, Part B.

[cit11] Shakeri Kardar Z., Beyki M. H., Shemirani F. (2016). Takovite-aluminosilicate@MnFe_2_O_4_ nanocomposite, a novel magnetic adsorbent for efficient preconcentration of lead ions in food samples. Food Chem..

[cit12] Ezoddin M., Shemirani F., Abdi Kh., Saghezchi M. K., Jamali M. R. (2010). Application of modified nano-alumina as a solid phase extraction sorbent for the preconcentration of Cd and Pb in water and herbal samples prior to flame atomic absorption spectrometry determination. J. Hazard. Mater..

[cit13] Afkhami A., Saber-Tehrani M., Bagheri H., Madrakian T. (2011). Flame atomic absorption spectrometric determination of trace amounts of Pb (ii) and Cr (ii) in biological, food and environmental samples after preconcentration by modified nano-alumina. Microchim. Acta.

[cit14] Esen C., Andac M., Bereli N., Say R., Henden E., Denizli A. (2013). Highly selective ion-imprinted particles for solid phase extraction of Pb^2+^ ions. Mater. Sci. Eng., C.

[cit15] Tajali Rad F., Kefayati H., Shariati Sh. (2019). Synthesis of propyl aminopyridine modified magnetite nanoparticles for cadmium(II) adsorption in aqueous solutions. Appl. Organomet. Chem..

[cit16] Enteshari Najafabadi M., Hayamian T. K., Hashemian Z. (2015). Aptamer-conjugated magnetic nanoparticles for extraction of adenosine from urine followed by electrospray ion mobility spectrometry. J. Pharm. Biomed. Anal..

[cit17] Hua X., Zhou Z., Yuan L., liu S. (2013). Selective collection and detection of MCF-7 breast cancer cells using aptamer-functionalized magnetic beads and quantum dots based nano-bio-probes. Anal. Chim. Acta.

[cit18] Shariati Sh., Faraji M., Yamini Y., Rajabi A. A. (2011). Magnetic nanoparticles modified with sodium dodecyl sulfate for removal of safranin O dye from aqueous solution. Desalination.

[cit19] Keyhanian F., Shariati Sh., Hesabi M. (2016). Magnetite nanoparticles with surface modification for removal of methyl violet from aqueous solution. Arabian J. Chem..

[cit20] Shariati S., Khabazipour M., Safa F. (2017). Synthesis and application of amine functionalized silica mesoporous magnetite nanoparticles for removal of chromium (VI) from aqueous solutions. J. Porous Mater..

[cit21] Toutounchi S., Shariati S., Mahanpoor K. (2019). Synthesis of nano-sized magnetite mesoporous carbon for removal of Reactive Yellow dye from aqueous solutions. Appl. Organomet. Chem..

[cit22] Huang C. C., Chen C. T., Shiang Y. C., Lin Z. H., Chang H. T. (2009). Synthesis of fluorescent carbohydrate-protected Au nanodots for detection of Concanavalin A and Escherichia coli. Anal. Chem..

[cit23] Song D., Yang R., Wang H., Li W., Wang H., Long H., Long F. (2017). A label-free SERRS-based nanosensor for ultrasensitive detection of mercury ions in drinking water and wastewater effluent. Anal. Methods.

[cit24] Musumeci D., Montesarchio D. (2012). Polyvalent nucleic acid aptamers and modulation of their activity: a focus on the thrombin binding aptamer. Pharmacol. Ther..

[cit25] Bock L. C., Griffin L. C., Latham J. A., Vermass E. H., Toole J. J. (1992). Selection of single-stranded DNA molecules that bind and inhibit human thrombin. Nature.

[cit26] Ellington A. D., Szostak J. W. (1990). In vitro selection of RNA molecules that bind specific ligands. Nature.

[cit27] Tuerk C., Gold L. (1990). Systematic evolution of ligands by exponential enrichment: RNA ligands to bacteriophage T4 DNA polymerase. Science.

[cit28] OleaJr C., Weidmann J., Dawson P. E., JoJce G. F. (2015). An L-RNA aptamer that binds and inhibits RNase. Chem. Biol..

[cit29] Pinto A., Polo P. N., Rubio M. J., Svobodova M., Lerga T. M., Osulliran C. K. (2016). Apta-PCR, methods. Mol. Biol..

[cit30] Sun H., Tan W., Zu Y. (2015). Aptamers: Versatile molecular recognition probes for cancer detection. Analyst.

[cit31] Madru B., Chapuis-Hugon F., Peyrin E., Pichon V. (2009). Determination of cocaine in human plasma by selective solid-phase extraction using an aptamer-based sorbent. Anal. Chem..

[cit32] Madru B., Chapuis-Hugon F., Pichon V. (2011). Novel extraction supports based on immobilized aptamers: evaluation for the selective extraction of cocaine. Talanta.

[cit33] White R., Rusconi C., Scardino E., Wolberg A., Lawson J., Hoffman M., Sullenger B. (2001). Generation of species cross-reaction aptamers using “toggle” SELEX. Mol. Ther..

[cit34] Jojce G. F. (1994). In vitro evolution of nucleic acids. Curr. Opin. Struct. Biol..

[cit35] Liu C., Huang C. C., Chang H. T. (2009). Highly selective DNA-based sensor for lead (ii) and mercury (ii) ions. Anal. Chem..

[cit36] Hense J. A., Wang J., Kawde A. N., Xiang Y., Gothelf K. V., Collins G. (2006). Quantum-Dot/Aptamer-based ultrasensitive multi-analyte electrochemical biosensor. J. Am. Chem. Soc..

[cit37] Hasanzadeh M., Shadju N., Guardia M. (2015). Iron and iron-oxide magnetic nanoparticles as signal-amplification elements in electro chemical biosensing, Trends. Anal. Chem..

[cit38] Divsar F., Habibzadeh K., Shariati Sh., Shahriarinour M. (2015). Aptamer conjugated silver nanoparticles for the colorimetric detection of arsenic ions using response surface methodology. Anal. Methods.

[cit39] Chun L., Cheng-Zhi H. (2014). Detection of lead ions in water based on the surface energy transfer between gold nanoparticles and fluorescent dyes. Chin. J. Anal. Chem..

[cit40] Taghdisi S. M., Sarreshtehdar Emrani S., Tabrizian K., Ramezani M., Abnous K., Sarreshtehdar Emrani A. (2014). Environ. Toxicol. Pharmacol..

[cit41] Rahnama S., Shariati Sh., Divsar F. (2018). Synthesis of functionalized magnetite titanium dioxide nanocomposite for removal of Acid fuchsine dye. Comb. Chem. High Throughput Screening.

[cit42] Tahmasebi E., Yamini Y. (2012). Facile synthesis of new nanosorbent for magnetic solid phase extraction by self assembling of bis-(2,4,4-trimethyl pentyl)-dithiophosphinic acid on Fe_3_O_4_@Ag core@shell nanoparticles: characterization and application. Anal. Chim. Acta.

[cit43] Sigel H. (1993). Interactions of metal ions with nucleotides and nucleic acids and their constituents. Chem. Soc. Rev..

[cit44] Sigel R. K., Sigel H. (2010). A stability concept for metal ion coordination to single-stranded nucleic acids and affinities of individual sites. Acc. Chem. Res..

[cit45] Izatt R. M., Christensen J. J., Rytting J. H. (1971). Sites and thermodynamic quantities associated with proton and metal ion interaction with ribonucleic acid, deoxyribonucleic acid, and their constituent bases, nucleosides, and nucleotides. Chem. Rev..

[cit46] Munshi A. M., Agarwal V., Ho D., Raston C. L. (2016). Magnetically directed assembly of nanocrystals for catalytic control of a three-component coupling reaction. Cryst. Growth.

[cit47] Amarjargal A., Tijing L. D., Im I.-T., Kim C. S. (2013). Simultaneous preparation of Ag/Fe_3_O_4_ core–shell nanocomposites with enhanced magnetic moment and strong antibacterial and catalytic properties. Chem. Eng. J..

[cit48] Wei S., Li J., He J., Zhao W., Wang F., Song X., Xu K., Wang J., Zhao C. (2020). Paper chip-based colorimetric assay for detection of Salmonella typhimurium by combining aptamer-modified Fe3O4@Ag nanoprobes and urease activity inhibition. Microchim. Acta.

[cit49] Link S., Wang Z. L., El-Sayed M. A. (1999). Alloy formation of gold-silver nanoparticles and the dependence of the plasmon absorption on their composition. J. Phys. Chem. B.

[cit50] Tao Z., Zhou Y., Duan N., Wang Z. (2020). A colorimetric aptamer sensor based on the enhanced peroxidase activity of functionalized graphene/Fe_3_O_4_-AuNPs for detection of lead(II) ions. Catalysts.

[cit51] Yu S. H., Lee C.-S., Kim T. H. (2019). electrochemical detection of ultratrace lead ion through attaching and detaching dna aptamer from electrochemically reduced graphene oxide electrode. Nanomaterials.

[cit52] Huang Y., Yang C., Sun Z., Zeng G., He H. (2015). Removal of cadmium and lead from aqueous solutions using nitrilotriacetic acid anhydride modified ligno-cellulosic material. RSC Adv..

[cit53] Cheng Y., Yang C., He H., Zeng G. (2016). Biosorption of Pb(II) ions from aqueous solution by waste biomass from biotrickling filters: kinetics, isotherms and thermodynamics. J. Environ. Eng..

[cit54] Ozcan S. G., Satiroglu N., Soylak M. (2010). Column solid phase extraction of iron(III), copper(II), manganese(II)
and lead(II) ions food and water samples on multi-walled carbon nanotubes. Food Chem. Toxicol..

[cit55] Xu H., Wu Y., Wang J., Shang X., Jiang X. (2013). Simultaneous preconcentration of cadmium and lead in water samples with silica gel and determination by flame atomic absorption spectrometry. J. Environ. Sci..

[cit56] Baghban N., Yilmaz E., Soylak M. (2017). Nanodiamond/MoS_2_ nanorod composite as a novel sorbent for fast and effective vortex-assisted micro solid phase extraction of lead(II) and copper(II) for their flame atomic absorption spectrometric detection. J. Mol. Liq..

[cit57] Ghanemi K., Nikpour Y., Omidvar O., Maryamabadi A. (2011). Sulfur-nanoparticle-based method for separation and preconcentration of some heavy metals in marine samples prior to flame atomic absorption spectrometry determination. Talanta.

